# Autoinflammation with infantile enterocolitis associated with a novel CARD domain mutation in *NLRC4*: a case report and literature review

**DOI:** 10.3389/fped.2026.1878056

**Published:** 2026-07-16

**Authors:** Yifan Mao, Yongmei Xie

**Affiliations:** 1 Department of Pediatric Gastroenterology, West China Second University Hospital of Sichuan University, Chengdu, China; Key Laboratory of Birth Defects and Related Diseases of Women and Children, Ministry of Education, Sichuan University, Chengdu, China; 2 West China College of Clinical Medicine, Sichuan University, Chengdu, China

**Keywords:** autoinflammation diseases, infliximab, NLRC4 gene, VEO-IBD, whole-exome sequencing

## Abstract

**Background:**

Autoinflammation with Infantile Enterocolitis (AIFEC, OMIM#616050) is a rare autosomal dominant autoinflammatory disorder caused by gain-of-function mutations in the NLRC4 gene. The disease is characterized by recurrent systemic inflammatory flares with enterocolitis as a hallmark feature, though clinical presentations are broad and often nonspecific.

**Case presentation:**

This report describes a noteworthy case of a pediatric patient with rare NLRC4 gene mutation leading to AIFEC. The patient was a 2-year-and-4-month-old female who presented with recurrent fever, chronic diarrhea, mucous bloody stools, and multi-system involvement. She had a history of multiple infections since early childhood. Laboratory investigations indicated significantly elevated inflammatory markers and endoscopic examination revealed multiple ulcers in the ileocolonic region. Whole-exome sequencing identified a *de novo* heterozygous mutation c.167A > G (p.His56Arg) in the CARD domain of NLRC4 gene, which was validated by Sanger sequencing and functionally assessed for pathogenicity. This specific gene locus mutation has not been reported in domestic or international literature to date. Clinical remission was achieved following infliximab treatment.

**Conclusion:**

This case identifies a novel NLRC4 variant (c.167A > G, p.His56Arg) located in the CARD domain. Our findings expand the known mutational and phenotypic spectrum of AIFEC and add to emerging clinical evidence suggesting the potential utility of infliximab as a targeted therapeutic option for this rare autoinflammatory condition.

## Introduction

The NLRC4 (NLR family CARD domain-containing protein 4) is a crucial intracellular pattern recognition receptor (PRR) that detects bacterial flagellin and components of the type III secretion system (1). Upon recognizing pathogen-associated molecular patterns (PAMPs), NLRC4 assembles into an inflammasome, driving the cleavage of pro-caspase-1 and the subsequent release of pro-inflammatory cytokines such as IL-1β and IL-18 and induces pyroptosis (2). Mutations in the NLRC4 gene lead to constitutive inflammasome activation, causing Autoinflammation with Infantile Enterocolitis (AIFEC, OMIM #616050), a rare autosomal dominant genetic disorder, first reported in 2014 (3). Typical clinical manifestations include severe diarrhea starting in the neonatal period accompanied by abnormally elevated serum IL-18 levels. In severe cases, it may present with symptoms of macrophage activation syndrome (MAS). Some patients may experience rashes, oral ulcers, joint swelling and pain.

To date, only 15 cases have been reported summarized in Table 3 (3, 6–14). All cases presented with unexplained fever, gastrointestinal symptoms, and elevated inflammatory markers. Most cases did not respond well to traditional immunosuppressants, and instead rely on cytokine-targeted therapy. The majority of documented NLRC4 mutations are localized within the NBD and HD1 domains. In contrast, mutations affecting the N-terminal caspase recruitment domain (CARD) remain exceedingly rare. The CARD domain is uniquely essential for direct downstream signalling, as it mediates homotypic CARD-CARD interactions with pro-caspase-1 or the adaptor ASC, thereby enabling inflammasome assembly and signal amplification (4). Therefore, mutations here may induce distinct patterns compared to other domains. This report describes a unique form of AIFEC with a novel heterozygous mutation (c.167A > G; p.His56Arg) in the CARD domain of NLRC4. By analyzing the clinical and genetic characteristics of the patient, along with a comprehensive literature review, we aim to refine the genotype-phenotype correlation of AIFEC and provide critical clinical insights into the management of refractory inflammasomopathies. Importantly, our experience demonstrates the utility of anti-TNF agents as an effective acute rescue therapy, while highlighting the vital necessity of early transition to upstream-targeted blockades for achieving sustained, long-term remission in refractory NLRC4-associated autoinflammation.

## Case presentation

A 2-year-and-4-month-old female was admitted to our hospital in May 2025 presenting with a one-month history of “recurrent fever, diarrhea, and refusal to walk”. Previous outpatient laboratory findings revealed a significantly elevated white blood cell (WBC) count, high-sensitivity C-reactive protein (hs-CRP), and erythrocyte sedimentation rate (ESR). After excluding infectious and other potential etiologies, she was diagnosed with systemic-onset juvenile idiopathic arthritis (sJIA) and responded transiently to prednisone acetate (5 mg, tid). However, two days prior to admission, unmonitored prednisone discontinuation triggered a severe recurrence of high fever (up to 39.2 °C), persistent diarrhea and hematochezia.

The patient is the first-born child of the family. She had documented allergies to milk and eggs, presenting with diarrhea. Since infancy, she had exhibited recurrent fever of unknown origin, chronic diarrhea with bloody mucoid stools, and multiple episodes of recurrent infections. No family history of inherited disorders was reported. Both parents were healthy while her grandfather had a history of chronic diarrhea.

**Figure 1:**
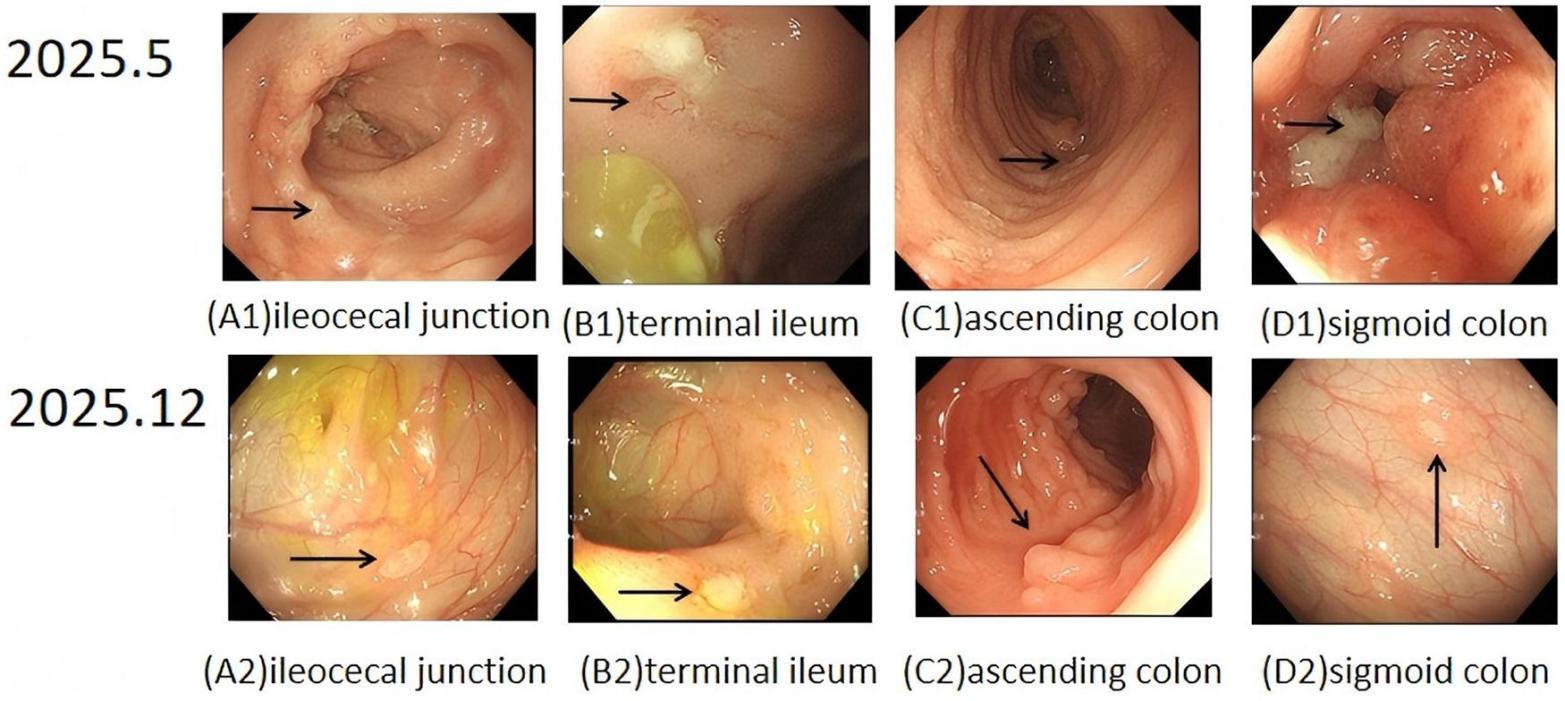
Colonoscopic images of the patient with AIFEC at two time points. Black arrows explicitly indicate the locations of mucosal ulcers, polypoid hyperplasia and Intestinal stenosis. The upper panel **(A1–D1)** captures endoscopic findings in May 2025 (patient aged 2 years and 4 months). **(A1)** Multiple polypoid hyperplasia are observed in the ileocecal junction. **(B1,C1)** Multiple circular ulcers are observed in the iterminal ileum, and ascending colon. **(D1)** Intestinal stenosis is observed in the sigmoid colon. The lower panel **(A2–D2)** shows the follow-up colonoscopy in December 2025 (patient aged 2 years and 11 months). **(A2,B2,D2)** Multiple circular ulcers are observed in the ileocecal junction, terminal ileum, and sigmoid colon. **(C2)** Multiple polypoid hyperplasia are observed in the ascending colon.

Based on her physical examinations at the time of admission, her weight was 9.6 kg (below the 3rd percentile, <P3). Bilateral conjunctival congestion and multiple enlarged cervical lymph nodes were noted, but no skin rash was observed. The oral mucosa was intact without ulcers. The abdomen was soft with a palpable liver edge 2 cm below the costal margin. Perianal and perineal skin erythema was noted without ulceration. Laboratory tests upon admission revealed the following: white blood cell count 2.6 × 10^9/L, the level of hs-CRP was 90.5 mg/L, and ESR of 41 mm/h. Stool culture detected Salmonella Typhi infection. Routine urinalysis, stool tests, liver and kidney function tests, humoral immunity panels, autoantibody screening, ANCA testing, tuberculosis investigations, and contrast-enhanced abdominal CT showed no significant abnormalities. The colonoscopy results indicated multiple ulcers in the terminal ileum, and colon, accompanied by partial intestinal stenosis and polypoid hyperplasia (Figure 1).

**Figure 2:**
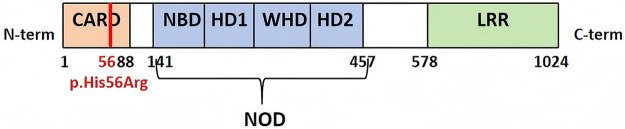
Structure of NLRC4 protein and the mutation sites. The NLRC4 protein consists of an N-terminal CARD (Cysteine aspartic acid protease activation and recruitment domain), a central NOD [Nucleotide-binding domain, Contains nucleotide-binding domain (NBD), helical domain (HD1, HD2), and winged helix domain (WHD)], and a C-terminal LRR (Leucine-rich repeat domain). The vertical red line marks the exact position of the novel *de novo* heterozygous mutation, c.167A > G (p.His56Arg), discovered within the functional CARD domain of our patient.

**Figure 3:**
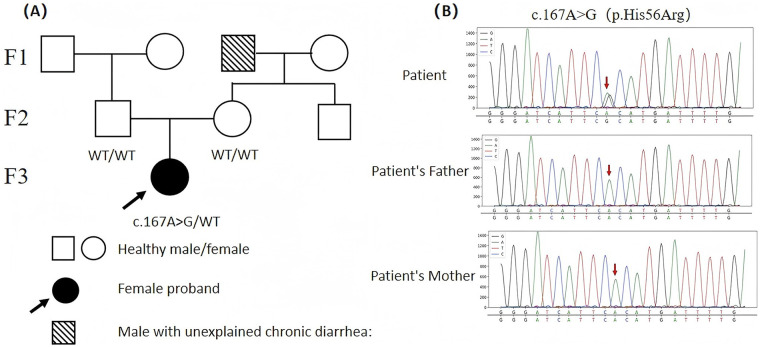
Panel **(A)** (pedigree). Pedigree chart of the proband with Autoinflammation with Infantile Enterocolitis (AIFEC). Squares represent males, circles represent females, and solid symbols indicate affected individuals. The black arrow points to the proband. Panel **(B)** (Sanger sequencing). Sanger sequencing results of the family. Red arrows indicate the targeted nucleotide substitution locus.

After admission, the patient was treated with antibiotics for infection treatment, along with lactobacillus acidophilus for intestinal microbiota modulation. Switching to an amino-acid-based formula milk did not resolve the mucoid bloody stools. Subsequent administration of prednisone acetate resolved fever and diarrhea. Given the infancy-onset, recurrent infections, refractory enterocolitis, poor response to antimicrobials but improvement with anti-inflammatory therapy, and prominent systemic inflammation, very-early-onset inflammatory bowel disease (VEO-IBD) secondary to an inborn error of immunity (IEI) was highly suspected. To further clarify the etiology, following parental consent, peripheral blood samples were obtained from the patient and both parents for whole-exome sequencing, which showed that the patient had a mutation c.167A > G, p.His56Arg: NM_001199138.2 in NLRC4, resulting in the substitution of histidine with arginine at residue 56 (Figure 2). Sanger sequencing confirmed that this mutation was absent in both parents (*de novo*), and this specific variant has not been previously reported. Based on the American College of Medical Genetics and Genomics (ACMG) guidelines (5), this variant was classified as likely pathogenic (PS2 + PM2_Supporting + PP4). The pedigree chart and Sanger sequencing validation are presented in Figure 3.

**Figure 4:**
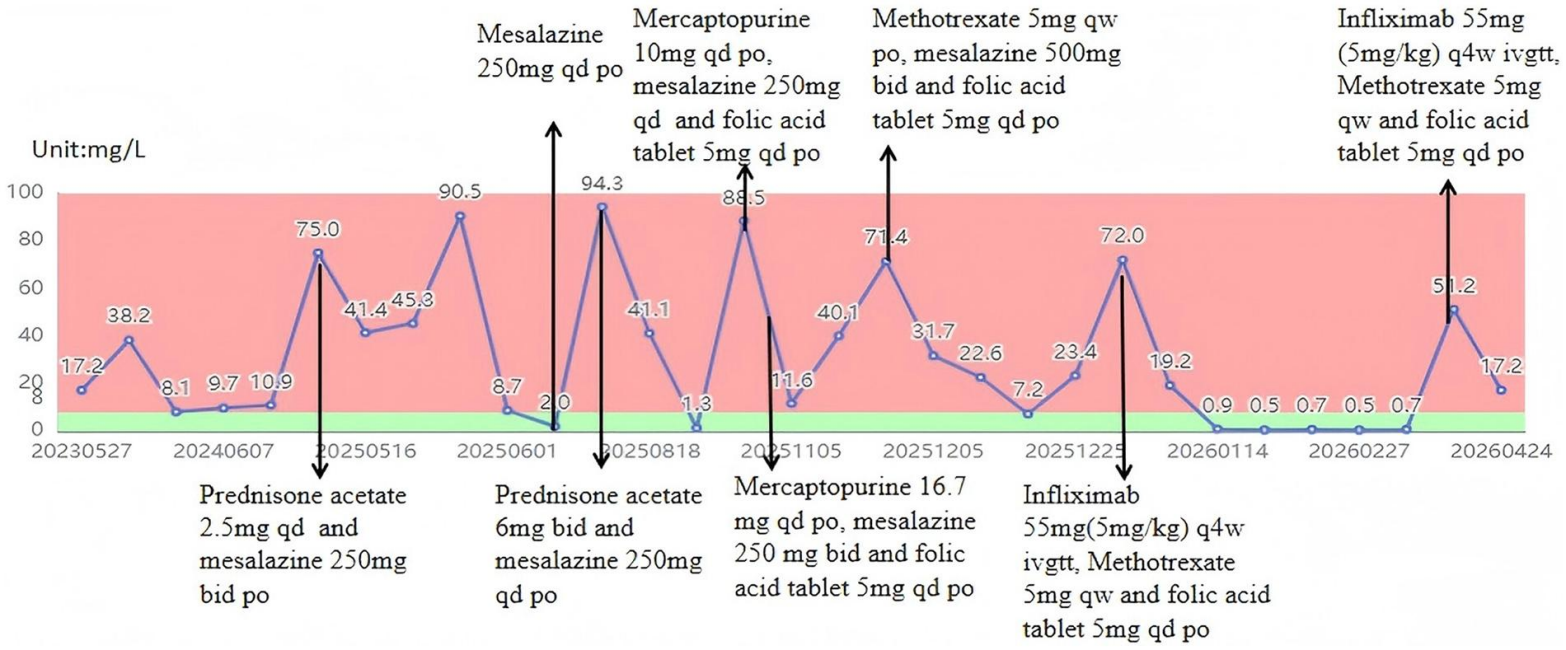
Longitudinal trajectory of serum C-reactive protein (CRP) levels and the corresponding therapeutic timeline of the patient. The green shaded area represents the normal reference range for CRP, while the red shaded area represents elevated pathological levels. The horizontal Axis tracks the step-by-step therapeutic interventions administered sequentially.

Two months later, the patient was readmitted due to recurrent fever lasting three days. Cytokine profiling revealed a profound hyperinflammatory state in Table 1: IL-6 at 29.07 pg/mL(reference:＜=20 pg/mL), IL-8 at 80.61 pg/mL(reference:＜=21.4 pg/mL), TNF-α at 15.86 pg/mL(reference:＜=5.50 pg/mL), and significantly elevated IFN-*γ* at 303.78 pg/mL(reference:＜=17.3 pg/mL). Interestingly, peripheral serum IL-1β levels remained within the normal range at 2.46 pg/mL (reference: ＜=12.1 pg/mL). Notably, due to acute presentation and technical constraints at the local facility, serum IL-18 levels could not be obtained at that time. During the next few months (from August 2025 to January 2026), despite temporary control with corticosteroids and mesalazine, sequential maintenance therapies with mercaptopurine and methotrexate failed. Follow-up colonoscopy revealed multiple ulcers involving the terminal ileum and colon, with the lesions being more severe than before (Figure 1). Given the suboptimal response to conventional immunosuppressants and the persistent severe mucosal inflammation, targeted biologic therapy was initiated with infliximab (a TNF-α inhibitor) 55 mg (5 mg/kg) every four weeks. Following the initial infusions, the patient exhibited a dramatic and rapid clinical response: fever resolved, stool normalized, and inflammatory markers plummeted, suggesting the efficacy of infliximab as an induction therapy (Figure 4). The patient was subsequently maintained on regular infliximab infusions. She remained in clinical remission for approximately three months. However, during the recent follow-up at the 3-month mark, the patient experienced a clinical relapse with recurrent fever and rebounded CRP levels (51.2 mg/L). Our test results showed that the serum concentration of infliximab was 1.7 ug/mL, and the serum level of anti-infliximab antibodies was less than 4 ng/mL. Given this clinical trajectory, the patient is currently under close monitoring, and transitioning to upstream targeted therapies, such as IL-1 or IL-18 blockade, is being actively evaluated for long-term maintenance in necessity.

**Table 1:** Cytokine test of the patient.

Examination Item	Result	Reference value	Unit
IL-1β	2.46	＜=12.1	pg/mL
IL-2	12.01	＜=11.40	pg/mL
IL-2R	953	＜=710	U/L
IL-6	29.07	＜=20	pg/mL
IL-8	80.61	＜=21.4	pg/mL
IL-10	18.83	＜=5.9	pg/mL
TNF-α	15.86	＜=5.50	pg/mL
IFN-*γ*	303.78	＜=17.3	pg/mL

This case highlights a severe, early-onset presentation of AIFEC driven by a novel CARD domain mutation. While anti-TNF therapy provided rapid acute rescue, the subsequent secondary loss of response indicates that downstream cytokine blockade may be insufficient for long-term maintenance in NLRC4-driven inflammasomopathies. The patient's medical treatment process is shown in Table 2.

**Table 2:** The condition and corresponding treatment timeline of the case patient.

Timeline	Symptoms	Treatments
2025.5.27	Fever, abdominal pain, diarrhea, bloody stools and arthralgia and joint swelling	Prednisone acetate 2.5 mg qd^a^ and mesalazine 250 mg bid^a^
2025.7.16	No symptoms	Mesalazine 250 mg qd
2025.8.16	Fever	Prednisone acetate 6 mg bid and mesalazine 250 mg qd
2025.10.15	No symptoms	Mercaptopurine 10 mg qd, mesalazine 250 mg qd and folic acid tablet 5 mg qd
2025.11.1	Fever, abdominal pain, diarrhea and bloody stools	Mercaptopurine 16.7 mg qd, mesalazine 250 mg bid and folic acid tablet 5 mg qd
2025.11.30	Fever and diarrhea	Methotrexate 5 mg qw^a^, mesalazine 500 mg bid and folic acid tablet 5 mg qd
2026.1.5	Fever, diarrhea and bloody stools	Infliximab 55 mg q4w^a^, Methotrexate 5 mg qw and folic acid tablet 5 mg qd
2026.4.14	Fever	Infliximab 55 mg q4w, Methotrexate 5 mg qw and folic acid tablet 5 mg qd

a qd: once daily, bid: twice daily, qw: quaque week; q4w: quaque 4 weeks.

## Literature review

Search the CNKI, Wanfang, VIP databases and PubMed, Web of Science databases with the keywords “autoinflammation with infantile enterocolitis”, “Autoinflammation diseases”, “NLRC4 gene”, and “AIFEC”. As of May 2026, only 14 clinically well-documented cases have been reported globally (3, 6–14). Including the present case, the total number reaches 15 patients harboring 10 distinct pathogenic variants, whose clinical characteristics are summarized in Table 3. All cases presented with unexplained fever, gastrointestinal symptoms, and elevated inflammatory markers, while secondary manifestations (e.g., skin rash, arthralgia/swelling, and oral ulcers) occurred infrequently.

**Table 3:** Summary of global AIFEC cases.

Case	Sex	Age	Mutation	Protein Variant	Domain	Clinical Manifestation	Family History	Treatment Regimen	Prognosis
Case1	F	2y4m	c.167A > G	p.His56Arg	CARD	Diarrhea, Abdominal pain, Bloody stools, Fever, Arthralgia and joint swelling	Parents not carriers	Prednisone acetate Mercaptopurine Methotrexate TNFα-blockade (Infliximab)	Improve
Case2 (3)	F	7y	c.1009A > T	p.Thr337Ser	HD1	Diarrhea, Fever, Rash	Parents not tested	Anakinra	Improve
Case3 (6)	F	1y	c.A91C	p.Asn31His	CARD	Diarrhea, Fever, Convulsions	Mother is a carrier	Methylprednisolone, TNFα-blockade (Adalimumab)	Improve
Case4 (6)	M	14y	c.1202T > C	p.Val401Ala	HD1	Bloody stools, Fever, Convulsions, Beading of middle cerebral artery, Oral ulcers	Parents not tested	Cyclophosphamide, Prednisone; Colchicine, TNFα-blockade (Adalimumab)	Improve
Case5 (6)	M	2y	c.390delG	p.Ser132Alafs*21	CARD-NBD	Diarrhea, Fever	Parents not tested	Prednisone, Azathioprine	Improve
Case6 (7)	M	3d	c.1021G > C	p.Val341Leu	HD1	Bloody stools, Fever, Aseptic meningitis, Rash	Parents not carriers	IVIG, Prednisone acetate	Death
Case7 (8)	M	12d	c.1021G > C	p.Val341Leu	HD1	Diarrhea, Bloody stools, Fever, Renal failure, Rash	Parents not tested	Methylprednisolone, Anakinra, Rapamycin	Improve
Case8 (9)	M	1w	c.1022T > C	p.Val341 Ala	HD1	Diarrhea, Fever	Father and half-brother affected	—	Death
Case9 (9)	M	5y	c.1022T > C	p.Val341 Ala	HD1	Enteritis, Fever, Renal failure, Myalgia	Father and half-brother affected	—	—
Case10 (9)	M	43y	c.1022T > C	p.Val341 Ala	HD1	Diarrhea, Fever, Arthralgia and joint swelling	Two sons affected	IVIG, Dexamethasone, Cyclosporine	Improve
Case11 (10)	M	20d	c.1022T > C	p.Val341 Ala	HD1	Diarrhea, Bloody stools, Fever, Perianal abscess	Parents not tested	Anakinra	Improve
Case12 (11)	F	6w	c.1022T > C	p.Val341 Ala	HD1	Diarrhea, Bloody stools, Fever, Rash	Parents not tested	rhIL-18BP	Improve
Case13 (12)	M	3d	c.1357C > T	p. Arg453*	NBD	Bloody stools, Distension, Shortened small intestine	Parents not tested	Surgery	Improve
Case14 (13)	-	20d	c.1010° C > A	p.Thr337Asn	HD1	Fever, Rash Pancytopenia	Parents not tested	Prednisone acetate, Cyclosporine, NI-0501	—
Case15 (14)	M	16m	c.1027A > C	p.Ile343Asn	HD1	Fever, Diarrhea, Vasculitic skin lesions	Parents not tested	Methylprednisolone, Cyclosporine, Anakinra, Emapalumab, FMT	—

## Discussion

### Pathogenesis and NLRC4 mutations

AIFEC is a rare autosomal dominant disorder caused by NLRC4 gene mutations that result in inflammasome hyperactivation. The condition primarily manifests during infancy and early childhood, presenting with diverse and nonspecific clinical features (15). Characterized by recurrent inflammatory episodes, its hallmark symptoms include periodic fever, secretory diarrhea, neonatal colitis, cutaneous eruptions, arthralgia/arthritis, and MAS. Additional manifestations may involve oral ulcerations, perianal abscesses, and renal or neurological involvement. Disease flares are commonly triggered by physical exhaustion, emotional distress, psychological stressors, and bacterial or viral infections (3). Relevant study has shown that the earlier the age of onset, the more severe the clinical symptoms will be (16).

The NLRC4 gene is located on chromosome 2p22.3 and consists of nine exons encoding a 1,024-amino acid protein (17). The NLRC4 protein comprises an N-terminal caspase recruitment domain (CARD), a central nucleotide-binding oligomerization domain (NOD), and a C-terminal leucine-rich repeat (LRR) domain. The NOD domain contains a nucleotide-binding domain (NBD), helical domains (HD1 and HD2), and a winged-helix domain (WHD) (18). Previous studies have identified that the majority of NLRC4 mutations are localized within the NBD and HD1 domains (3, 6–14). These mutations typically destabilize the autoinhibitory state of the protein, allowing spontaneous oligomerization without ligand binding (19). In contrast, our study revealed a novel NLRC4 mutation (c.167A > G, p.His56Arg) situated in the CARD domain. The CARD domain is functionally unique because it bypasses the regulatory machinery of the NOD domain and directly mediates homotypic CARD-CARD interactions with downstream pro-caspase-1 or the adaptor protein ASC to drive signal amplification (4). Mechanistically, we hypothesize that this specific mutation alters the local conformation, significantly enhancing the spontaneous oligomerization affinity of the CARD domain and leading to ligand-independent recruitment of pro-caspase-1 or ASC. This represents a distinct pathogenic mechanism wherein the mutation drives autonomous, ligand-independent downstream assembly. Further studies may require the use of animal models for functional validation. Furthermore, this genotypic divergence critically correlates with distinct phenotypic manifestations. While conventional NBD/HD1 mutations frequently precipitate catastrophic MAS, both our patient and the previously reported CARD-mutant peer manifested a clinical phenotype heavily polarized toward intractable, early-onset enterocolitis (AIFEC) with severe, localized mucosal ulceration (6). This phenotypic contrast implies a tissue-specific vulnerability: CARD-domain hyperactivation may exert a disproportionately potent pathological impact on intestinal epithelial cells, driving localized pyroptosis and mucosal barrier breakdown, rather than driving unchecked systemic immune destruction.

A unique feature of our case was the concurrent Salmonella typhi infection. Under physiological conditions, the wild-type NLRC4 inflammasome can be activated by bacterial flagellin and components of the flagellar-associated secretion system (20). In the context of the NLRC4 CARD mutation may lower the energy barrier for inflammasome activation and place it in a ligand-independent, hyper-primed state. It is highly plausible that the invasion of Salmonella typhi acted as an environmental trigger utilizing a ligand-dependent pathway (flagellin) to induce a devastating cascading amplification upon the already sensitized NLRC4 pathway, which may amplify inflammasome activation and exacerbate clinical severity (21).

### Biological and clinical implications of the cytokine profile

Furthermore, intestinal epithelial cells continuously produce mature IL-18 (22), which serves as a crucial diagnostic indicator for differentiating NLRC4-associated autoinflammatory diseases from other inflammasomopathies, particularly NLRP3-mediated disorders. Due to technical limitations, IL-18 levels were not measured in our patient. Although peripheral serum IL-1β levels were not markedly elevated, this does not preclude intense local inflammasome activation. IL-1β has a notably short half-life in systemic circulation and primarily exerts its destructive effects locally within the intestinal mucosa. Furthermore, the profound elevation of downstream cytokines, including IL-6, IL-8, and IFN-*γ*, and TNF-α suggests that a transient or localized IL-1β/IL-18 pulse had already robustly initiated the systemic inflammatory cascade.

### Clinical and therapeutic implications of TNF blockade

Although the canonical consequence of NLRC4 inflammasome hyperactivation is the excessive cleavage and release of IL-1β and IL-18 (2), our patient demonstrated a remarkable clinical response to infliximab, a TNF-α inhibitor. This therapeutic efficacy can be explained by the downstream inflammatory cascade initiated by pyroptosis. NLRC4 is highly expressed in intestinal epithelial cells and macrophages (23), triggering secondary macrophage activation and the massive release of broader pro-inflammatory cytokines, including TNF-α. Elevated TNF-α plays a critical role in mediating mucosal ulceration and maintaining the vicious cycle of intestinal inflammation in AIFEC (24). As a classical chimeric human-murine IgG1 monoclonal antibody, the profound potency of infliximab stems not only from its high-affinity neutralization of soluble TNF-α but also from its specific binding to transmembrane TNF-α (tmTNF) on the surface of target cells. This binding process triggers reverse signaling and precisely induces the apoptosis of pathologically activated T cells and macrophages locally through robust antibody-dependent cellular cytotoxicity (ADCC) and complement-dependent cytotoxicity (CDC) (25). By depleting the population of inflammatory cells at the source, infliximab effectively interrupted the downstream cytokine storm and mitigated localized intestinal damage by blocking TNF-α (26). This clinical observation provides vital mechanistic insight and aligns closely with recent findings from an animal model of NLRC4-associated autoinflammation developed by Wang et al. (17), which revealed that TNF blockade can significantly ameliorate infantile enterocolitis pathology. Furthermore, this therapeutic rationale is perfectly corroborated by existing clinical literature: as summarized in Table 3, the only other reported patient with a CARD domain mutation (Case 3) also achieved clinical remission following the administration of Adalimumab, another anti-TNF-α agent (6). This parallel evidence solidifies the indispensable value of anti-TNF-α therapies as an acute rescue strategy for AIFEC flares.

Notably, infliximab effectively controlled the acute hyperinflammatory response. However, the patient experienced disease relapse, manifesting as fever and elevated CRP after three months of therapy. Secondary loss of response to biologic agents is a well-documented phenomenon (27). Potential mechanisms include the development of anti-drug antibodies that reduce drug bioavailability and persistent IL-1β/IL-18 overproduction due to NLRC4 inflammasome mutations that bypass TNF-α blockade. The patient presented with low infliximab trough levels without detectable anti-drug antibodies, which was attributable to the persistent upstream overactivation of the NLRC4 inflammasome, with continuous excessive production of IL-1β and IL-18 bypasses downstream TNF-α blockade. For this we may increase doses of induction therapy to 10 mg/kg or changing the method of intravenous administration to subcutaneous injection to maintain trough levels (28). Additionally, the comprehensive and systemic blockade of TNF-α inevitably causes profound disruption to the body's immune surveillance and defense mechanisms. There is an urgent need to adopt therapeutic drug monitoring (TDM) and individualized concomitant immunosuppressant regimens.

This case underscores that while anti-TNF therapy is effective for managing acute autoinflammatory flares (AIFEC), sustained remission may necessitate upstream targeting of IL-1 or IL-18 signaling pathways. Future strategies may transfer to upstream IL-1/IL-18 blockade (e.g., rhIL-18BP) (11). If these prove insufficient, alternative targeted therapies such as JAK1 inhibitors or rapamycin could be explored based on emerging evidence (18).

### Limitations and future directions

This study has several limitations. First, due to technical constraints during the patient's acute admissions, serum IL-18 levels were not quantified. And retrospective measurement of IL-18 mRNA was unfeasible due to the lack of cryopreserved peripheral blood mononuclear cells (PBMCs) from the acute phase, representing a descriptive gap in our laboratory profile. Second, functional assays to directly validate the *in vitro* pathogenic mechanism of the novel c.167A > G mutation were not performed. Future functional and mechanistic studies are warranted to fully elucidate how this specific CARD domain mutation drives autonomous inflammasome propagation. Finally, as a single case report, conclusions regarding the long-term efficacy of infliximab should be interpreted cautiously. Future multicenter studies with larger cohorts and functional validations are needed to better characterize genotype-phenotype correlations and establish standardized targeted treatment protocols for NLRC4-related AIFEC.

## Conclusion

In conclusion, we report a patient with a *de novo* NLRC4 variant (c.167A > G p.His56Arg) in CARD domain. This is the first report of this specific mutation associated with AIFEC, suggesting that infliximab may serve as an effective acute rescue therapy for severe AIFEC flares. However, secondary loss of response at three months highlights the need for upstream targeted therapies, such as IL-1/IL-18 blockade, for long-term disease maintenance. It provides new insights for investigating disease pathogenesis, improving clinical diagnosis and treatments. Given the rarity of NLRC4-associated AIFEC and current limited sample sizes, clear genotype-phenotype correlations remain to be established. Future multicenter studies with larger cohorts are needed to better characterize the clinical and genetic features of NLRC4-related AIFEC, develop effective therapies, and enhance clinical recognition of this disorder.

## Data Availability

The original contributions presented in the study are included in the article/Supplementary Material, further inquiries can be directed to the corresponding author.

## References

[1] Wang Y Lingappa S Kang Z. Current understanding of the NLRC4 inflammasome in autoinflammation and enterocolitis. Commun Biol. (2026) 9(1):611. 10.1038/s42003-026-10174-3 42082718 PMC13149970

[2] Egan MS Zhang J Shin S. Human and mouse NAIP/NLRC4 inflammasome responses to bacterial infection. Curr OpinMicrobiol. (2023) 73:102298. 10.1016/j.mib.2023.102298 PMC10225321 37058933

[3] Canna SW de Jesus AA Gouni S Brooks SR Marrero B Liu Y. An activating NLRC4 inflammasome mutation causes autoinflammation with recurrent macrophage activation syndrome. Nat Genet. (2014) 46(10):1140–6. 10.1038/ng.3089 25217959 PMC4177369

[4] Fu J Schroder K Wu H. Mechanistic insights from inflammasome structures. Nat Rev Immunol. (2024) 24(7):518–35. 10.1038/s41577-024-00995-w 38374299 PMC11216901

[5] Richards S Aziz N Bale S Bick D Das S Gastier-Foster J. Standards and guidelines for the interpretation of sequence variants: a joint consensus recommendation of the American College of Medical Genetics and Genomics and the Association for Molecular Pathology. Genet Med. (2015) 17(5):405–24. 10.1038/gim.2015.30 25741868 PMC4544753

[6] Asna Ashari K Parvaneh N Mirnia K Ayati M Saeedi M Salehzadeh F. Three cases of autoinflammatory disease with novel NLRC4 mutations, and the first mutation reported in the CARD domain of NLRC4 associated with autoinflammatory infantile enterocolitis (AIFEC). Pediatr Rheumatol Online J. (2024) 22(1):90. 10.1186/s12969-024-01020-z 39425177 PMC11487858

[7] Cheng P Sun HQ Yu ZY Li MC Li LF. A case of neonatal-onset autoinflammation with infantile enterocolitis caused bya de novo NLRC4 gene variation[J]. Chinese Journal of Perinatal Medicine. (2022) 25(12):968–70. 10.3760/cma.j.cn113903-20220520-00512

[8] Barsalou J Blincoe A Fernandez I Dal-Soglio D Marchitto L Selleri S. Rapamycin as an adjunctive therapy for NLRC4 associated macrophage activation syndrome. Front Immunol. (2018) 9:2162. 10.3389/fimmu.2018.02162 30319625 PMC6166634

[9] Romberg N Al Moussawi K Nelson-Williams C Stiegler AL Loring E Choi M. Mutation of NLRC4 causes a syndrome of enterocolitis and autoinflammation. Nat Genet. (2014) 46(10):1135–9. 10.1038/ng.3066 25217960 PMC4177367

[10] Siahanidou T Nikaina E Kontogiorgou C Tzanoudaki M Stefanaki K Skiathitou AV. Autoinflammation with infantile enterocolitis associated with recurrent perianal abscesses. J Clin Immunol. (2019) 39(3):237–40. 10.1007/s10875-019-00611-w 30864118

[11] Canna SW Girard C Malle L de JA Romberg N Kelsen J. Life-threatening NLRC4-associated hyperinflammationsuccessfully treated with IL-18 inhibition. J Allergy Clin Immunol. (2017) 139(5):1698–701. 10.1016/j.jaci.2016.10.022 27876626 PMC5846100

[12] Wang X Xiao Y Ge T Zhang T Li X. Autoinflammation with infantile enterocolitis induced by a heterozygous variant (c.1357C>T) in the NLRC4 gene: a case report. Front Pediatr. (2026) 14:1822554. 10.3389/fped.2026.1822554 42290740 PMC13254022

[13] Bracaglia C de Graaf K Pardeo M Lapeyre G Ferlin W Nelson R. Anti interferon-gamma (IFN*γ*) monoclonal antibody treatment in a patient carrying an NLRC4 mutation and severe hemophagocytic lymphohistiocytosis. Pediatric Rheumatology. (2015) 13(Suppl 1):O68. 10.1186/1546-0096-13-S1-O68

[14] Bracaglia C Marucci G Del Chierico F Russo A Pardeo M Pires Marafon D. Microbiota transplant to control inflammation in a patient with NLRC4 gain-of-function-induced disease. J Allergy Clin Immunol. (2023) 152(1):302–3. 10.1016/j.jaci.2023.03.031 37178069

[15] Ionescu D Peñín-Franch A Mensa-Vilaró A Castillo P Hurtado-Navarro L Molina-López C. First description of late-onset autoinflammatory disease due to somatic NLRC4 mosaicism. Arthritis Rheumatol. (2022) 74(4):692–9. 10.1002/art.41999 34672126

[16] Wang Y Gao JZ Gurung P Short SP Xiong Y Canna SW. An animal model of NLRC4-associated autoinflammation and infantile enterocolitis reveals novel therapeutic strategies. Cell Mol Immunol. (2025) 22(12):1567–80. 10.1038/s41423-025-01355-x 41116055 PMC12660311

[17] Duncan JA Canna SW. The NLRC4 inflammasome. Immunol Rev. (2018) 281(1):115–23. 10.1111/imr.12607 29247997 PMC5897049

[18] Cahill S Humphries F. Inflammasomopathies: mechanisms and disease signatures. TrendsImmunol. (2025) 46(5):372–85. 10.1016/j.it.2025.03.008 40263090

[19] Kitamura A Sasaki Y Abe T Kano H Yasutomo K. An inherited mutation in NLRC4 causes autoinflammation in human and mice. J Exp Med. (2014) 211(12):2385–96. 10.1084/jem.20141091 25385754 PMC4235634

[20] Naseer N Zhang J Bauer R Constant DA Nice TJ Brodsky IE. Salmonella enterica serovar Typhimurium induces NAIP/NLRC4- and NLRP3/ASC-independent, caspase-4-dependent inflammasome activation in human intestinal epithelial cells. Infect Immun. (2022) 90(7):e0066321. 10.1128/iai.00663-21 35678562 PMC9302179

[21] Hou C Wang Z Eichenberger V Martinon F. Dysregulation of inflammasomes in autoinflammatory diseases. Joint Bone Spine. (2025) 92(5):105903. 10.1016/j.jbspin.2025.105903 40194758

[22] Shimizu M Takei S Mori M Yachie A. Pathogenic roles and diagnostic utility of interleukin-18 in autoinflammatory diseases. Front Immunol. (2022) 13:951535. 10.3389/fimmu.2022.951535 36211331 PMC9537046

[23] Tong G Shen Y Li H Qian H Tan Z. NLRC4, Inflammation and colorectal cancer (review). Int J Oncol. (2024) 65(4):99. 10.3892/ijo.2024.5687 39239759 PMC11387119

[24] Souza RF Caetano MAF Magalhães HIR Castelucci P. Study of tumor necrosis factor receptor in the inflammatory bowel disease. World J Gastroenterol. (2023) 29(18):2733–46. 10.3748/wjg.v29.i18.2733 37274062 PMC10237104

[25] Horiuchi T Mitoma H Harashima S Tsukamoto H Shimoda T. Transmembrane TNF-alpha: structure, function and interaction with anti-TNF agents. Rheumatology (Oxford). (2010) 49(7):1215–28. 10.1093/rheumatology/keq031 20194223 PMC2886310

[26] Kumar M Murugesan S Ibrahim N Elawad M Al Khodor S. Predictive biomarkers for anti-TNF alpha therapy in IBD patients. J Transl Med. (2024) 22(1):284. 10.1186/s12967-024-05058-1 38493113 PMC10943853

[27] Singh S Murad MH Fumery M Sedano R Jairath V Panaccione R. Comparative efficacy and safety of biologic therapies for moderate-to-severe Crohn's Disease: a systematic review and network meta-analysis. Lancet Gastroenterol Hepatol. (2021) 6(12):1002–14. 10.1016/S2468-1253(21)00312-5 34688373 PMC8933137

[28] Eldredge JA Kaur R Chua CW Chidgey T Tano M Singh N. Subcutaneous infliximab use in children with inflammatory bowel disease in an Australian center. J Pediatr Gastroenterol Nutr. (2026) 82(2):439–46. 10.1002/jpn3.70267 41251027

